# Eye Tracking in Parkinson’s Disease: A Review of Oculomotor Markers and Clinical Applications

**DOI:** 10.3390/brainsci15040362

**Published:** 2025-03-31

**Authors:** Pierluigi Diotaiuti, Giulio Marotta, Francesco Di Siena, Salvatore Vitiello, Francesco Di Prinzio, Angelo Rodio, Tommaso Di Libero, Lavinia Falese, Stefania Mancone

**Affiliations:** 1 Department of Human Sciences, Society and Health, University of Cassino and Southern Lazio, 03043 Cassino, Italy; giulio.marotta@unicas.it (G.M.); francesco.disiena@unicas.it (F.D.S.); salvatore.vitiello@unicas.it (S.V.); a.rodio@unicas.it (A.R.); tommaso.dilibero@unicas.it (T.D.L.); l.falese@unicas.it (L.F.); s.mancone@unicas.it (S.M.); 2 Department of Human Sciences, Philosophy and Education, University of Salerno, 84084 Fisciano, Italy; fdiprinzio@unisa.it

**Keywords:** Parkinson’s disease, eye tracking, saccadic dysfunction, fixation instability, pupillary response, cognitive impairment, machine learning, neurorehabilitation

## Abstract

(1) Background. Eye movement abnormalities are increasingly recognized as early biomarkers of Parkinson’s disease (PD), reflecting both motor and cognitive dysfunction. Advances in eye-tracking technology provide objective, quantifiable measures of saccadic impairments, fixation instability, smooth pursuit deficits, and pupillary changes. These advances offer new opportunities for early diagnosis, disease monitoring, and neurorehabilitation. (2) Objective. This narrative review explores the relationship between oculomotor dysfunction and PD pathophysiology, highlighting the potential applications of eye tracking in clinical and research settings. (3) Methods. A comprehensive literature review was conducted, focusing on peer-reviewed studies examining eye movement dysfunction in PD. Relevant publications were identified through PubMed, Scopus, and Web of Science, using key terms, such as “eye movements in Parkinson’s disease”, “saccadic control and neurodegeneration”, “fixation instability in PD”, and “eye-tracking for cognitive assessment”. Studies integrating machine learning (ML) models and VR-based interventions were also included. (4) Results. Patients with PD exhibit distinct saccadic abnormalities, including hypometric saccades, prolonged saccadic latency, and increased anti-saccade errors. These impairments correlate with executive dysfunction and disease progression. Fixation instability and altered pupillary responses further support the role of oculomotor metrics as non-invasive biomarkers. Emerging AI-driven eye-tracking models show promise for automated PD diagnosis and progression tracking. (5) Conclusions. Eye tracking provides a reliable, cost-effective tool for early PD detection, cognitive assessment, and rehabilitation. Future research should focus on standardizing clinical protocols, validating predictive AI models, and integrating eye tracking into multimodal treatment strategies.

## 1. Introduction

Parkinson’s disease (PD) is a chronic, progressive neurodegenerative disorder primarily affecting dopaminergic neurons in the substantia nigra. This degeneration leads to motor dysfunctions such as bradykinesia, rigidity, and resting tremor [1]. However, beyond these hallmark motor symptoms, non-motor impairments significantly impact patients’ quality of life. Among these, cognitive decline, executive dysfunction, visual disturbances, spatial memory, and language function have gained increasing attention in recent years [2,3].

Emerging evidence suggests that eye movement abnormalities are among the earliest detectable neurological deficits in PD, preceding some of the more commonly recognized motor impairments [4]. These deficits include alterations in saccadic eye movements, fixation stability, smooth pursuit tracking, and pupillary responses, all of which are linked to dysfunctions in cortico-basal ganglia loops and other neural pathways involved in oculomotor control [5]. Given that PD pathology extends beyond the basal ganglia to include brainstem structures such as the superior colliculus and frontal eye fields, disruptions in these circuits likely contribute to the oculomotor disturbances observed in affected individuals [4,5,6,7].

These disruptions in eye movement of Parkinson’s patients are closely linked to the degeneration of the dopaminergic system, which affects the basal ganglia and frontal eye fields, both critical for voluntary eye movements. The altered saccades, in particular, involve difficulties in accurately shifting gaze towards a target, often resulting in incomplete eye movements that require compensatory corrective saccades.

These oculomotor dysfunctions are not only indicative of motor impairments but also show a significant relationship with cognitive dysfunction, particularly in executive control and attention. In addition to these well-known eye movement deficits, the relationship between ocular and manual coordination, or eye–hand coupling, is of great interest in PD. Although our review focuses on the eye-tracking measures of eye movement abnormalities, it is important to recognize how these deficits extend to broader motor activities. Alterations in visual processing, such as delayed or impaired saccades, have been shown to affect eye–hand coordination, impairing the ability to accurately reach and grasp objects, a crucial task for daily functioning in PD patients. This connection between eye movement and motor actions highlights the relevance of eye tracking as a tool for assessing the broader functional impairments in PD.

In recent years, eye-tracking technology has gained considerable interest as a non-invasive tool for assessing oculomotor function in neurological disorders. Unlike traditional clinical assessments, which rely on subjective observation, eye tracking provides high-precision data that can be analyzed in real time, allowing researchers and clinicians to monitor subtle changes in eye movements that may serve as early biomarkers of disease onset and progression [8].

Numerous studies have explored the potential of eye tracking in PD research, particularly for early detection, differential diagnosis, and disease monitoring. Given that eye movement impairments are closely linked to cognitive and motor dysfunctions, tracking these deficits over time may provide valuable insights into neurodegenerative trajectories and help predict cognitive decline in patients with PD [8,9]. The integration of eye-tracking data with artificial intelligence (AI) and machine learning algorithms has the potential to enhance diagnostic accuracy, facilitating personalized treatment approaches [10].

Oculomotor disturbances in PD, including hypometric saccades, prolonged saccadic latency, and fixation instability, are not only important for diagnostic purposes but also offer significant implications for therapeutic interventions. Recent studies suggest that eye-tracking technology can be used to monitor the effectiveness of various treatments, such as dopaminergic therapy [9], cognitive rehabilitation [11], and visual–motor training [11]. Emerging therapies integrating virtual reality (VR) and biofeedback aim to enhance oculomotor function and eye–hand coordination, potentially improving both motor and cognitive outcomes in PD patients [11,12]. As such, eye tracking could play a pivotal role in the development of personalized rehabilitation strategies tailored to the specific needs of PD patients, offering a more targeted approach to treatment [13].

This narrative review synthesizes the current literature on eye movement impairments in Parkinson’s disease, focusing on saccadic abnormalities, fixation instability, smooth pursuit deficits, and pupillary changes. We discuss how these impairments correlate with disease severity, cognitive dysfunction, and neurophysiological alterations. We also emphasize the clinical implications of eye-tracking technology for both diagnosis and therapeutic intervention. We also explore emerging trends in AI-driven analysis, virtual reality-based rehabilitation programs, and the integration of eye tracking into multimodal neurophysiological assessments.

Through a comprehensive examination of existing studies, this review underscores the importance of eye movement analysis as a key component in PD research, advocating for the broader implementation of eye-tracking technologies in both clinical practice and experimental investigations. By establishing standardized assessment protocols and leveraging technological advancements, eye tracking could play a pivotal role in shaping the future of Parkinson’s disease management. It offers a promising avenue for early detection, progression monitoring, and therapeutic innovation.

In contrast to previous reviews that have focused primarily on diagnostic or cognitive aspects of oculomotor dysfunction in Parkinson’s disease, the present review offers a broader synthesis that includes motor–cognitive interactions, therapeutic implications, and innovative applications of emerging technologies such as AI-based analysis and VR-integrated rehabilitation. Several previous reviews have addressed aspects of oculomotor function in Parkinson’s disease. For example, Liao et al. [2] and Gibbs et al. [6] provided overviews of eye movement abnormalities with a focus on diagnostic potential. Others, such as Tsitsi et al. [14], have examined specific parameters like fixation duration and pupillary size in clinical populations. However, these reviews often focus on isolated features or specific clinical applications.

In contrast, the present work offers a comprehensive and integrative synthesis of the literature on saccadic impairments, fixation instability, smooth pursuit deficits, and pupillary dynamics, all assessed through eye tracking in PD. It also expands on prior work by discussing the role of machine learning, the clinical and therapeutic implications of these markers, and their relevance to rehabilitation strategies. This broader perspective highlights how eye tracking is evolving from a diagnostic tool to a potential instrument for monitoring disease progression and guiding personalized interventions. This comprehensive perspective aims to bridge the gap between clinical observations and technological innovation, providing a more holistic understanding of how eye-tracking metrics can inform both diagnosis and treatment.

## 2. Methods

This narrative review aims to synthesize and discuss the existing literature on eye movement abnormalities in Parkinson’s disease (PD) and explore the potential applications of eye-tracking technology for early diagnosis, disease monitoring, and rehabilitation. The literature search was performed between September 2024 and January 2025, focusing on peer-reviewed studies available at that time. Given the complexity of oculomotor dysfunction in PD, this review integrates findings from clinical studies, experimental research, and technological advancements, providing a comprehensive and interpretative synthesis of current knowledge rather than a systematic meta-analysis.

The literature included in this review was selected based on its relevance to the key themes of the study, specifically focusing on saccadic impairments, fixation instability, smooth pursuit deficits, and pupillary changes in PD. Studies exploring the clinical and technological applications of eye tracking in PD were also prioritized, with particular attention to early detection, disease progression monitoring, and neurorehabilitation strategies. The selection of articles was guided by thematic relevance rather than rigid inclusion or exclusion criteria, as the objective was to critically discuss diverse perspectives and emerging trends in the field.

Relevant studies were identified through structured searches in PubMed, Scopus, Web of Science, and Google Scholar, using tailored keyword combinations and Boolean operators (AND, OR) to optimize the search results. The search strategies applied were as follows: (1) PubMed: (“Parkinson’s disease” OR “PD”) AND (“eye-tracking” OR “oculomotor” OR “saccadic” OR “fixation instability” OR “smooth pursuit” OR “pupillary response”); (2) Scopus/Web of Science: TITLE-ABS-KEY (“Parkinson’s disease” AND “eye movements”) AND (“saccades” OR “fixation” OR “oculomotor” OR “eye-tracking” OR “pupil”); (3) Google Scholar: “eye-tracking in Parkinson’s disease” + “saccadic latency” + “fixation instability” + “cognitive impairment”. Additional articles were retrieved through citation tracking, particularly from review papers and key experimental studies in the field.

**Figure 1:**
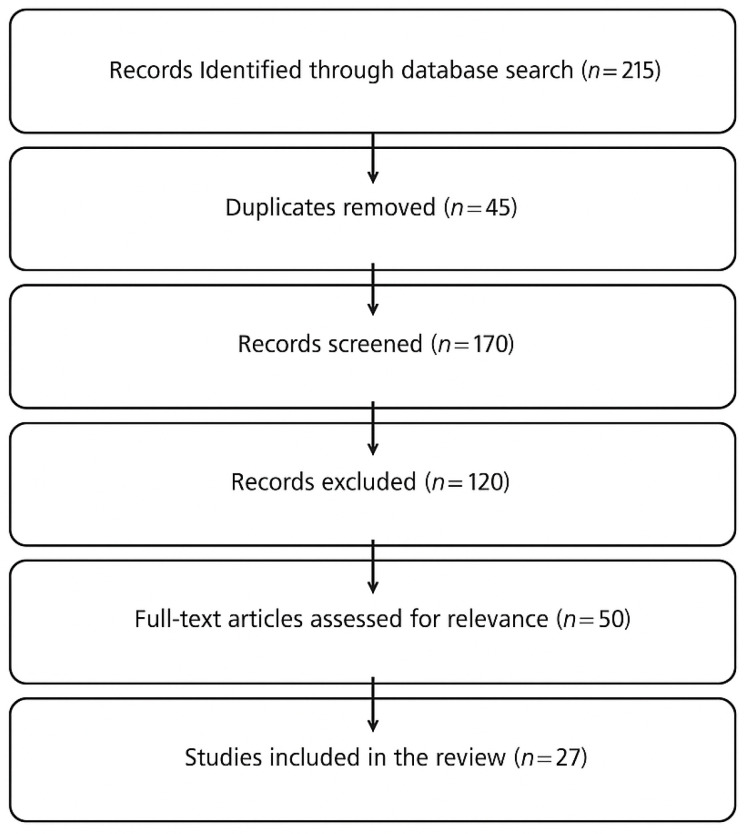
Flowchart illustrating the literature selection process for the narrative review. A total of 215 records were identified, 45 duplicates were removed, and 27 studies were included based on thematic relevance.

The discussion is structured around the primary oculomotor deficits observed in PD, analyzing how these impairments correlate with cognitive and motor dysfunction and how eye-tracking metrics can be leveraged for diagnostic and rehabilitative purposes. Given the increasing role of artificial intelligence (AI) in neurodegenerative disease research, studies employing machine learning models for eye-tracking analysis in PD were also reviewed to highlight innovative diagnostic approaches. Particular attention was given to studies that demonstrate the clinical feasibility of eye tracking in PD and those that propose future research directions for integrating eye tracking into clinical practice. A flowchart illustrating the number of records identified, screened, and included in the review is presented in Figure 1, in accordance with the narrative review design. While no formal inclusion/exclusion protocol was applied, studies were selected based on thematic relevance and quality of content.

## 3. Results

In order to better contextualize the findings presented in this section, Table 1 below summarizes the 27 major studies that we extracted from our empirical literature review on eye tracking in PD. These studies, ordered thematically and chronologically, offer insights into saccadic abnormalities, fixation instability, smooth pursuit deficits, and pupillary dysfunctions. The following sections summarize the main findings across the reviewed studies, grouped according to key oculomotor domains.

**Figure 2:**
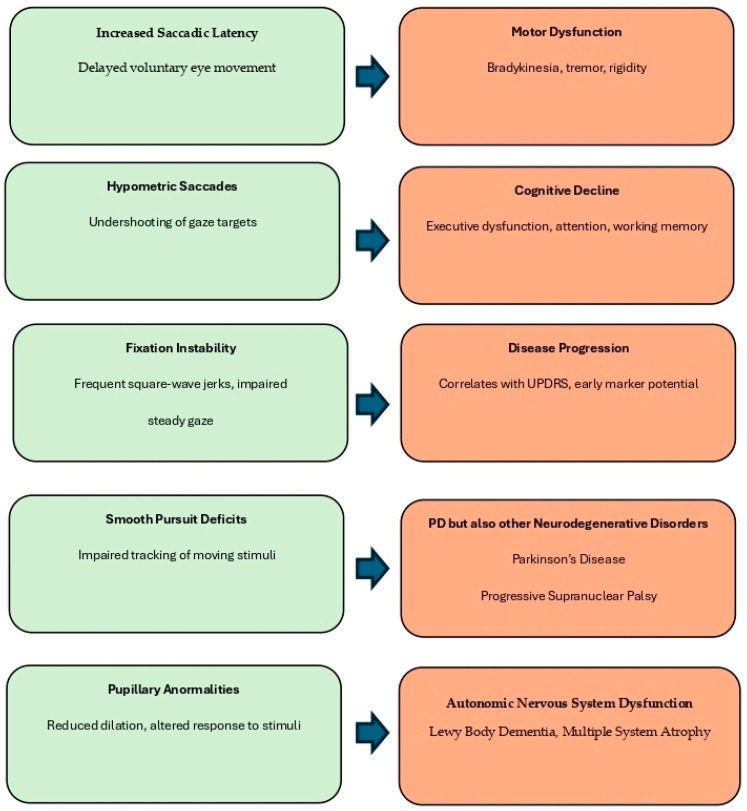
Summary of oculomotor alterations observed in Parkinson’s disease (PD) and their clinical relevance. Eye movement abnormalities, including increased saccadic latency, hypometric saccades, fixation instability, smooth pursuit deficits, and pupillary abnormalities, reflect dysfunctions across distinct neural circuits. These features are associated with various functional outcomes and disease markers.

Figure 2 provides a visual summary of the principal eye movement abnormalities observed in Parkinson’s disease and their associations with motor and cognitive impairment.

Saccadic abnormalities (delayed and hypometric eye movements) and fixation instability (e.g., square-wave jerks) are commonly linked to motor dysfunction and cognitive decline, particularly executive dysfunction and working memory deficits. Smooth pursuit deficits, characterized by impaired tracking of moving stimuli, are associated not only with PD but also with other neurodegenerative disorders such as progressive supranuclear palsy (PSP) and Huntington’s disease, reflecting cerebellar and basal ganglia involvement. Pupillary abnormalities, such as reduced dilation and delayed light response, are indicative of autonomic nervous system dysfunction and are frequently seen in Lewy Body Dementia (LBD) and Multiple System Atrophy (MSA), where dysautonomia is a prominent clinical feature. Together, these oculomotor signs may contribute to disease staging, differential diagnosis, and the identification of early cognitive or motor deterioration in PD and atypical parkinsonian syndromes

### 3.1. Eye Movement Abnormalities in Parkinson’s Disease

#### 3.1.1. Saccadic Dysfunction in PD

Patients with Parkinson’s disease (PD) exhibited significant increases in saccadic latency, with an average delay of 200 ms (SD = 25 ms) compared to healthy controls (mean = 140 ms, SD = 15 ms) [4,8,15]. This increase in latency was strongly correlated with disease progression, as measured by UPDRS motor scores (r = 0.72, *p* < 0.001), indicating that saccadic latency may serve as a useful biomarker for monitoring motor decline in PD.

Saccades are rapid, ballistic eye movements that serve to reposition the fovea, the central part of the retina responsible for high-acuity vision, toward new visual stimuli. These movements are essential for visual scanning, reading, and object localization, and their proper execution depends on a complex interplay between cortical, subcortical, and brainstem structures. In Parkinson’s disease (PD), the degeneration of dopaminergic pathways, particularly in the substantia nigra and basal ganglia, leads to dysfunctional saccadic control, resulting in various forms of saccadic impairments [11]. Among these, hypometric saccades are one of the most frequently observed abnormalities, characterized by reduced amplitude, meaning the eyes do not fully reach the intended target, requiring corrective saccades to compensate for incomplete movements [16]. This phenomenon is closely linked to impaired motor initiation and disrupted feedback processing in the basal ganglia, which plays a critical role in controlling voluntary movement. Another common impairment is increased saccadic latency, where PD patients exhibit slower initiation times, leading to delayed responses to visual stimuli. This suggests functional deficits in the fronto-striatal-thalamic circuit, which is responsible for movement planning and execution.

Beyond these deficits in saccadic precision and initiation, PD patients also exhibit square-wave jerks, which are involuntary, small-amplitude eye movements that occur between fixations, disrupting visual stability. These micro-movements have been associated with abnormal inhibitory control mechanisms in the superior colliculus and cerebellum, structures involved in fixation stability and saccadic suppression [17]. PD affects voluntary goal-directed saccades more than reflexive saccades, meaning that stimulus-driven saccades tend to be less impaired than internally guided saccades [18]. This reflects preferential deficits in executive control mechanisms, particularly those regulated by the prefrontal cortex and basal ganglia circuits [19,20].

Numerous studies have examined saccadic dysfunction in PD using pro-saccade and anti-saccade tasks, which assess the ability to generate rapid, goal-directed eye movements and inhibit inappropriate responses [21,22,23]. Pro-saccade tasks require participants to shift their gaze toward a suddenly appearing stimulus, and PD patients often exhibit prolonged reaction times and hypometric saccades, indicating slower sensorimotor processing and disrupted motor output. Anti-saccade tasks, on the other hand, require individuals to suppress a reflexive saccade toward a stimulus and instead look in the opposite direction. PD patients frequently demonstrate increased error rates, reduced accuracy, and prolonged reaction times, which suggest deficits in inhibitory control and executive function, processes mediated by the frontal–striatal network. Hindle et al. and Archibald et al. [23,24] found significant correlations between saccadic performance and cognitive function in PD, particularly in tasks requiring working memory and executive control, reinforcing the idea that oculomotor dysfunction in PD extends beyond motor impairments and reflects underlying cognitive decline [25].

Further evidence from functional neuroimaging and electrophysiology has shown that saccadic impairments are associated with reduced activity in the frontal cortex, basal ganglia, and superior colliculus, supporting the idea that oculomotor deficits serve as biomarkers for deterioration in motor and cognitive functions in PD [26,27]. Given that saccadic abnormalities often emerge early in the disease, eye movement analysis holds significant diagnostic potential. Measuring saccadic latency, accuracy, and error rates could provide quantifiable markers of disease progression, offering a non-invasive, objective alternative to traditional motor assessments [28]. As research continues to refine machine learning algorithms and AI-driven eye-tracking models, integrating oculomotor biomarkers into clinical diagnostics and disease monitoring could greatly enhance early detection, stratification of disease subtypes, and targeted therapeutic interventions in Parkinson’s disease.

#### 3.1.2. Fixation Instability and Microsaccadic Intrusions

Fixation instability, measured by the frequency of square-wave jerks, increased with the severity of the disease. Early PD patients showed an average of 5.4 square-wave jerks per minute (SD = 2.1), while advanced PD patients exhibited a significantly higher average of 12.3 jerks per minute (SD = 3.4) (*p* = 0.03) [16,25]. This increase was correlated with cognitive decline, as assessed by the MoCA (r = −0.60, *p* < 0.01), suggesting that fixation instability could serve as an early indicator of motor and cognitive deterioration in PD.

Fixation is a fundamental oculomotor function that allows the eyes to maintain a steady gaze on a target, ensuring visual clarity, spatial stability, and efficient cognitive processing. In healthy individuals, fixation is supported by a delicate balance between involuntary micro-movements (microsaccades) and active control mechanisms, preventing visual fading while maintaining gaze precision [29]. In Parkinson’s disease (PD), fixation stability is significantly compromised, leading to involuntary eye drifts, excessive microsaccades, and frequent square-wave jerks. PD patients often struggle to maintain steady gaze and exhibit a significantly higher frequency of square-wave jerks, whose intrusions disrupt gaze fixation by shifting the eyes away from the target and then rapidly correcting back [30,31]. Shaikh et al. [16] proposed that fixation instability in PD contributes to broader cognitive and attentional deficits, as visual fixation plays a crucial role in maintaining spatial awareness, processing visual information, and sustaining attention. The presence of excessive microsaccades and square-wave jerks can lead to visual discomfort, impaired reading fluency, difficulties in processing complex visual scenes, exacerbating cognitive strain and functional impairment, suggesting deficits in oculomotor inhibitory control mechanisms. PD patients display abnormally frequent and dysregulated microsaccadic movements, which interfere with reading, fine visual discrimination, and attentional focus, making daily tasks more difficult [17].

Fixation control is governed by a network of cortical and subcortical structures, including the superior colliculus (SC), which plays a key role also in reflexive gaze control and visual attention, the frontal eye fields (FEFs), responsible for voluntary eye movement planning, and the basal ganglia, particularly the substantia nigra pars reticulata, which modulates saccadic suppression and fixation control. Krauzlis et al. [30] demonstrated that fixation control involves a dynamic interaction between these regions, with dopaminergic dysfunction in PD disrupting their functional connectivity, leading to deficits in voluntary gaze stabilization, increased oculomotor noise, and decreased ability to suppress involuntary eye movements, which causes instability in visual tracking.

Fixation instability has profound implications for PD patients, significantly affecting daily life activities. Frequent microsaccadic intrusions disrupt text tracking, leading to slower reading speeds and impaired comprehension. Unstable fixation may interfere with sustained attention in tasks requiring detailed visual examination, such as driving, object recognition, and facial identification, further reducing functional independence. Another critical consequence of fixation instability is its potential link to postural instability; impaired gaze fixation control has been associated with poorer balance and gait disturbances, which may contribute to an increased risk of falls and reduced mobility in PD patients [29,31,32].

Given the clinical significance of fixation instability, eye-tracking technology has emerged as a valuable tool for assessing and monitoring fixation impairments in PD. By quantifying fixation duration, microsaccadic frequency, and square-wave jerk rates, researchers and clinicians can gain objective insights into disease progression and its impact on cognitive and motor function. Eye-tracking assessments allow for the differentiation of PD from other neurodegenerative disorders with overlapping motor symptoms, such as progressive supranuclear palsy (PSP), which presents distinct patterns of fixation instability [32]. These tools can help track disease progression and serve as a biomarker for cognitive function, providing valuable information on executive dysfunction and attentional impairments in PD patients [2]. As advancements in machine learning and AI-driven analytics continue to refine eye-tracking methodologies, these tools could be further integrated into routine neurological assessments, allowing for earlier detection, personalized treatment strategies, and improved monitoring of disease progression.

#### 3.1.3. Smooth Pursuit and Convergence Deficits

Smooth pursuit eye movements are continuous, coordinated movements that allow the eyes to track a moving object smoothly rather than through discrete jumps (saccades). This function is essential for visual stability, motion perception, and spatial awareness in everyday activities, such as driving, reading, and navigating dynamic environments. In Parkinson’s disease (PD), smooth pursuit movements are often compromised, resulting in the delayed, fragmented, and inefficient tracking of moving stimuli. Several impairments have been identified in PD patients, including reduced pursuit gain, where the eyes lag behind moving targets, necessitating frequent catch-up saccades to compensate for tracking deficits. PD patients often rely on increased compensatory saccades to maintain fixation on a moving object, which disrupts the fluidity of gaze control and makes it harder to track motion continuously [16]. Another common issue is compromised binocular convergence, where individuals with PD experience difficulty focusing on near objects, a condition known as convergence insufficiency, which affects depth perception, spatial judgment, and near-field vision clarity, leading to difficulties in tasks such as reading and object manipulation [11].

Smooth pursuit movements are controlled by a complex network of brain regions, including the parieto-occipital cortex, which processes motion stimuli, the cerebellum, which fine-tunes the velocity and precision of pursuit movements, the frontal eye fields (FEF) and the basal ganglia. In PD, dopaminergic depletion in the basal ganglia disrupts the interaction between these regions, leading to deficits in pursuit initiation and maintenance. Dysfunction in the superior colliculus and brainstem pathways further impairs motion processing and gaze stabilization mechanisms, contributing to jerky, inefficient pursuit movements [5,30].

These impairments have significant functional consequences, affecting visual perception, motor coordination, and cognition. Difficulties in reading arise because tracking lines of text smoothly becomes challenging, leading to fatigue, slower reading speeds, and comprehension difficulties. Impaired motion perception can cause PD patients to struggle with accurately judging moving objects, which in turn affects spatial orientation and balance [33,34,35]. Navigation difficulties also emerge due to a reduced ability to track moving pedestrians, vehicles, or environmental cues, which increases the risk of falls and accidents [36,37]. Fooken et al. [20] found that PD patients required significantly more corrective saccades to maintain smooth pursuit, leading to disruptions in visual tracking accuracy, placing additional cognitive and motor demands on the individual and potentially contributing to fatigue and visual discomfort.

Binocular convergence results in blurred near vision, making activities such as reading and writing more difficult. In more severe cases, patients experience double vision (diplopia), which affects spatial coordination and increases fall risk. This misalignment also leads to increased eye strain and visual fatigue, as the brain struggles to compensate for conflicting binocular input [11]. Given the diagnostic and functional relevance of smooth pursuit and convergence abnormalities in PD, eye-tracking technology provides a valuable tool for assessing and monitoring these impairments. By quantifying smooth pursuit gain and compensatory saccades, clinicians can help differentiate PD from other neurodegenerative disorders, while tracking convergence ability over time can serve as an indicator of disease progression and cognitive decline.

#### 3.1.4. Pupillary Abnormalities and Cognitive Correlates

Pupillary responses are regulated by autonomic nervous system activity and cognitive processing demands, with pupil size dynamically modulated by sympathetic and parasympathetic pathways. The locus coeruleus–norepinephrine system (LC-NE) plays a crucial role in cognitive arousal, attentional control, and executive functioning [38]. In Parkinson’s disease (PD), disruptions in both autonomic regulation and neurocognitive processing manifest as pupillary dysfunctions, which may serve as biomarkers for disease progression and cognitive decline. Several deficits have been identified in PD patients, including a reduced baseline pupil size, indicative of parasympathetic overactivity and reduced sympathetic tone, possibly reflecting neurodegenerative changes in autonomic control circuits. Another key deficit is the altered pupillary reflex under stable lighting, pointing to brainstem dysfunction affecting autonomic regulation [39,40]. PD patients frequently exhibit shorter fixation durations, linked to dysfunctional gaze control and increased microsaccadic intrusions, which may contribute to visual fatigue, attentional lapses, and reading difficulties [14,25]. Tsitsi et al. [14] demonstrated that pupillary changes in PD correlate with both motor symptoms and cognitive impairments, reinforcing the idea that oculomotor biomarkers could provide insights into the broader neurodegenerative process.

The underlying neurophysiological mechanisms responsible for pupillary abnormalities in PD involve multiple brain regions that govern autonomic regulation and cognitive processing. Among these, the locus coeruleus (LC) modulates arousal, attention, and cognitive flexibility through norepinephrine signaling, while the superior colliculus (SC) integrates visual, motor, and autonomic functions, and the prefrontal cortex (PFC) plays a critical role in cognitive effort and executive decision making. The degeneration of the LC-NE system leads to dysregulated autonomic output and cognitive control impairments, resulting in abnormal pupillary responses that could reflect early neurodegenerative changes affecting these key regulatory pathways [38].

Beyond autonomic impairments, pupillary responses are closely linked to cognitive function in PD, with research suggesting that reduced pupil dilation during cognitive tasks is associated with slower information processing and executive dysfunction [23,38]. Pupil dynamics during attention and memory tasks have been found to predict early cognitive decline in PD patients, highlighting their potential as an early biomarker of neurodegeneration. Aberrant pupillary responses appear more pronounced in PD patients with mild cognitive impairment (MCI) and may serve as a precursor to dementia, further supporting the hypothesis that pupillometry could serve as a non-invasive physiological marker of neurocognitive deterioration [23,36,37].

### 3.2. Eye Tracking as a Diagnostic and Monitoring Tool in PD

#### 3.2.1. Correlation Between Eye Movements and Progression in Parkinson’s Disease

Eye movement abnormalities in Parkinson’s disease (PD) are increasingly recognized as early indicators of cognitive decline and disease progression. Oculomotor dysfunction, including saccadic impairments, fixation instability, and smooth pursuit deficits, correlates with executive dysfunction, attentional deficits, and working memory impairments in PD patients [23,24,25,26]. As neurodegeneration progresses, these deficits tend to worsen over time. Longitudinal studies have investigated the relationship between oculomotor function and cognitive decline in PD, demonstrating that saccadic parameters can predict disease progression [6,14,25]. Notably, Stuart et al. [8] followed PD patients for 54 months and found that shorter saccadic latency predicted more rapid cognitive deterioration.

The interplay between cognitive and motor functions in PD is well established: eye movements are closely linked to executive function and working memory through frontal-striatal circuits involving the dorsolateral prefrontal cortex (DLPFC), the supplementary eye field (SEF), and the basal ganglia. Dopaminergic depletion in these areas leads to both motor and cognitive impairments. Saccadic deficits serve as a proxy for broader neurodegenerative changes due to mechanisms such as prefrontal cortex dysfunction causing increased variability in saccadic execution.

Given their longitudinal stability and objective nature, oculomotor metrics have potential as standardized biomarkers for PD progression. Regular saccadic testing could help track cognitive and motor decline over time. Identifying patients with rapidly worsening oculomotor function could aid in stratifying individuals at higher risk for developing Parkinson’s disease dementia (PDD). Measuring changes in ocular–motor parameters following pharmacological or rehabilitative interventions can offer valuable insights into the effectiveness of various treatment strategies. By leveraging this technology for early detection and personalized management strategies tailored to motor and cognitive aspects of PD, researchers can improve patient outcomes significantly by enhancing diagnostic precision [2,14,38].

#### 3.2.2. Machine Learning Applications for Automated Diagnosis

Recent advancements in artificial intelligence (AI) and machine learning (ML) have revolutionized biomedical diagnostics, offering automated, objective, and high-precision analysis of complex neurological data. In the context of Parkinson’s disease (PD), AI-driven approaches are increasingly being explored to analyze eye-tracking data for early detection, continuous disease monitoring, and differential diagnosis. Eye movement abnormalities often manifest early in the disease course; thus, AI-based analysis of oculomotor biomarkers, such as saccadic latency, fixation instability, smooth pursuit deficits, and pupillary responses, can enhance diagnostic accuracy and improve patient stratification [39,40].

Machine learning models excel in pattern recognition and have been effective in detecting subtle alterations in eye movements that might precede noticeable clinical symptoms. These approaches allow for the identification of oculomotor dysfunctions before traditional motor symptoms emerge and facilitate differentiation between PD and other neurodegenerative disorders like progressive supranuclear palsy (PSP) and Multiple System Atrophy (MSA). Continuous eye-tracking assessments provide quantitative measures that help track disease progression over time, offering valuable insights into neurodegeneration trajectories [41]. Bredemeyer et al. [42] applied machine learning algorithms to predict disease severity based on oculomotor parameters across different PD stages.

Traditional PD diagnosis relies heavily on clinical examination and subjective symptom reporting, which can lead to variability and delays. AI-driven eye-tracking analysis offers precise metrics that reduce reliance on subjective assessments. Features like fixation duration, saccadic velocity, and pupillary dynamics provide biomarker-level accuracy for standardized diagnostic criteria. Automated classification is another significant advancement; machine learning models trained on large datasets enable clinician-independent classification of PD cases.

AI-based diagnostic tools assist neurologists by generating real-time probability scores for PD presence and severity. This capability is critical for early detection years before significant motor symptoms appear, a window allowing for earlier intervention and potentially slowing disease progression, and improves diagnostic specificity by distinguishing PD-related eye movement abnormalities from those seen in other diseases like PSP where vertical gaze dysfunction is an early hallmark.

Beyond diagnostic improvements, AI-powered eye-tracking analysis is scalable and cost-effective compared to conventional techniques such as MRI PET scans, making it suitable for widespread implementation, including home-based monitoring, reducing the healthcare burden. Various machine learning models, including supervised unsupervised deep learning approaches, offer unique advantages, each enhancing diagnostic precision when integrating multimodal data sources combining oculomotor neuroimaging genetic information [43,44].

### 3.3. The Role of Eye Tracking in Cognitive Assessment

#### 3.3.1. Eye Movements and Executive Function in PD

Eye movements are increasingly recognized as a window into the broader cognitive impairments associated with Parkinson’s disease (PD), particularly executive dysfunction, which encompasses deficits in attention, inhibitory control, and working memory. Executive function relies on the integrity of the frontal-striatal circuitry, a network affected by progressive neurodegeneration in PD. As the disease advances, impairments manifest not only in motor symptoms but also in cognitive deficits that can be objectively assessed through oculomotor tasks. Research has demonstrated that saccadic eye movements are highly sensitive to executive dysfunction in PD, making them valuable indicators of disease progression and cognitive decline [45].

Liao et al. [2] investigated the relationship between saccadic performance and cognitive impairments in PD. They found higher error rates in anti-saccade tasks due to deficits in inhibitory control, a core aspect of executive function regulated by the dorsolateral prefrontal cortex (DLPFC) and basal ganglia pathways. Increased saccadic latency correlated with slower cognitive processing speeds, indicating delays in goal-directed decision making linked to frontal-striatal dysfunction.

The connection between oculomotor function and executive processing highlights PD’s dual nature as both a movement disorder and a cognitive disorder. Since executive dysfunction often predates severe cognitive decline and Parkinson’s disease dementia (PDD), detecting early changes through eye-tracking technology offers opportunities for early diagnosis, risk stratification, and monitoring of disease progression [6,14].

Beyond diagnostic implications, studying executive dysfunction through eye movements offers potential therapeutic applications. Targeted interventions focusing on improving response inhibition may enhance both oculomotor control and cognitive function. Emerging evidence suggests benefits from computer-based training incorporating real-time eye-tracking feedback could improve executive function early on, potentially slowing its decline [46,47]. This growing body of evidence underscores adopting a multimodal approach considering both motor–cognitive trajectories for improved clinical outcomes using tools like those proposed by Gibbs et al. and Tsitsi [6,48].

#### 3.3.2. Visual Search and Reading Impairments

Patients with Parkinson’s disease (PD) often face significant challenges in visual exploration and reading fluency due to oculomotor impairments that disrupt text processing and the navigation of visually complex environments. Reading requires the coordination of fixation stability, saccadic control, and visual contrast sensitivity, functions disrupted in PD by degeneration in frontal-striatal circuits and associated oculomotor pathways. As a result, text scanning becomes inefficient; PD patients frequently lose their place or experience visual fatigue due to reduced fixation stability, characterized by involuntary eye drifts, microsaccadic intrusions, and square-wave jerks [48,49]. Prolonged saccadic latency further disrupts reading rhythm by delaying the initiation of eye movements. Normal reading involves precise sequences of saccades and fixations to smoothly traverse lines of text. However, in PD, saccades are often hypometric and delayed, requiring additional corrective movements that increase cognitive load and reduce reading efficiency.

Impairments in visual contrast sensitivity also affect letter recognition as PD patients struggle with distinguishing characters under low-contrast conditions [29,49]. These deficits are linked to dopaminergic dysfunction, affecting the retinal and cortical visual processing centers [30]. Given the impact on daily life, there is an urgent need for standardized eye-tracking assessments to evaluate cognitive impairments in PD objectively. Gibbs et al. [6] emphasized that incorporating objective eye-tracking tasks into clinical evaluations as traditional neuropsychological assessments may not fully capture subtle but disabling visual deficits experienced by PD patients.

### 3.4. Implications for Early Diagnosis and Clinical Interventions

#### 3.4.1. Potential for Early Diagnosis and Disease Monitoring

Eye-tracking technology holds significant potential as a diagnostic biomarker for early detection and continuous disease monitoring in Parkinson’s disease (PD). Unlike traditional clinical assessments, eye tracking provides an objective, quantifiable, and non-invasive method to detect subtle oculomotor dysfunctions before more pronounced motor symptoms emerge [2]. As PD progresses, cognitive and motor dysfunctions become increasingly apparent; measuring saccadic latency, fixation stability, smooth pursuit efficiency, and pupillary responses can serve as valuable markers for disease onset and progression [6]. Specific oculomotor signatures unique to PD, such as hypometric saccades, increased square-wave jerks, and prolonged anti-saccade reaction times, allow clinicians to differentiate PD from other neurodegenerative disorders, like progressive supranuclear palsy (PSP), Multiple System Atrophy (MSA), and Alzheimer’s disease [40].

Bek et al. [7] proposed that predictive models based on eye movement metrics could enable earlier intervention strategies by identifying preclinical PD biomarkers. Leveraging machine learning algorithms with large-scale datasets can facilitate risk stratification and individualized treatment plans before irreversible neurodegeneration occurs. Longitudinal studies could monitor disease progression while assessing therapeutic intervention effectiveness over time. Integrating eye tracking into clinical trials could provide real-time measures of therapeutic response by modulating certain oculomotor deficits through dopaminergic replacement therapy or neurorehabilitation programs [50]. Incorporating this technology into routine assessments has the potential to redefine early PD diagnosis by reducing diagnostic delays.

#### 3.4.2. Integration of Eye Tracking in PD Rehabilitation

The integration of eye-tracking technology into neurorehabilitation programs for Parkinson’s disease (PD) represents a promising approach to addressing oculomotor, cognitive, and motor impairments. Training programs focusing on gaze control, visual exploration, and motor–cognitive coordination could enhance functional outcomes and quality of life by targeting fixation instability, saccadic dysfunction, and impaired smooth pursuit, common challenges faced by PD patients [51]. One potential avenue involves training gaze control to improve reading fluency and visual exploration. Many PD patients struggle with reduced fixation stability, increased microsaccadic intrusions, and prolonged saccadic latencies, disrupting their ability to scan text and efficiently navigate complex environments. Using real-time eye-tracking feedback, patients can stabilize fixation, optimize saccadic efficiency, and reduce involuntary gaze shifts, improving reading speed comprehension and spatial navigation. Structured saccadic exercises could enhance motor–cognitive coordination, as PD patients often exhibit difficulties initiating controlling sequencing voluntary saccades. Training precise goal-directed movements may help strengthen neural pathways involved in movement execution and executive control, potentially counteracting the frontal-striatal dysfunction associated with PD [52,53].

A particularly exciting development is integrating eye tracking into virtual reality (VR)-based neurorehabilitation programs. VR environments combined with biofeedback create engaging adaptive rehabilitation experiences, allowing PD patients to practice real-world tasks in controlled settings. For instance, VR simulations guide patients through visual search tasks, spatial navigation challenges, and dynamic eye–hand coordination exercises, all while tracking adjusting individual oculomotor patterns. These interventions are beneficial in enhancing attentional flexibility, improving depth perception, and reducing the cognitive load associated with visual–motor integration. VR-based training helps reinforce compensatory strategies by teaching individuals to adapt their behavior in response to real-world challenges, such as crossing streets, scanning shelves, and following social cues [10,11,12].

**Table 1:** Eye-tracking studies in Parkinson’s disease.

	Thematic Area	Autors	Year	Sample	Objective	Methods	Results	Key Findings
1	Smooth Pursuiit Deficits	Tanabe, J., Tregellas, J., Miller, D., Ross, R. G., & Freedman, R.	2002 [34]	PD patients (N = 48, 26 M/22 F)	Study brain activation during smooth pursuit	Neuroimaging smooth pursuit tasks	Disrupted pursuit control mechanisms	Neurophysiological basis of pursuit deficits
2	Fixation Instability	Shaikh, A.G., Xu-Wilson, M., Grill, S., & Zee D. S.	2011 [16]	PD patients (N = 40, 22 M/18 F)	Investigate ‘staircase’ square-wave jerks in early PD	Oculomotor testinf	Increased square-wave jerks in early PD	Fixation instability as an early PD marker
3	Fixation instability	Marx, S., Respondek, G., Stamaleou, M., Dowiasch, S., Stoll, J., Bremmer, F., & Einhäuser, W.	2012 [32]	PD patients (N = 50, 30 M/20 F)	Differentiate PSP from PD using fixation analysis	Mobile eye.tracking	Distinct fixation instability patterns in PSP vs PD	Eye-tracking helps differentitate neurodegenerative disorders
4	Pupillary abnormalities	Wang, C.A., & Munoz, D. P.	2015 [39]	PD patients (N = 52, 30 M/22 F)	Analyze cognitive modulation of pupil size	Pupillometry and neurocognitive tasks	Dysregulates autonomic control	Pupillary changes correlate with cognitive decline
5	Reading and Visual Impairments	Ekker, M. S., Janssen, S., Seppi, K., Poewe, W., de Vries, N. M., Theelen, T., & Bloem, B. R.	2017 [54]	PD patients (N = 50, 27 M/23 F)	Analyze ocular disorders in PD	Comprensive visual assessments	High prevalence of visual deficits	Ocular disorders often overlooked in PD
6	Fixation Instability	Wong, O. W., Chan, A. Y., Wong, A., Lau, C. K., Yeung, J. H., Mok, V. C., ... & Chan, S.	2018 [25]	PD patients (N = 40, 20 M/20 F)	Examine eye movement parameters and cognitive function	Eye-tracking with cognitive assessments	Fixation instability correlates with cognitive decline	Oculomotor measures predict neurocognitive impairment
7	Eye-tracking in PD Cognitive Assessment	Luke, S. G., Darowski, E. S., & Gale, S. D.	2018 [49]	PD patients (N = 40, 20 M/20 F)	Predict cognitive impairments through eye traking	Eye movement tasks	Correlation between eye movements and cognitive decline	Early cognitive impairment detection
8	Reading and Visual Impairments	Jehangir, N., Yu, C. Y., Song, J., Shariati, M. A., Binder, S., Beyer, J., ... & Liao, Y. J.	2018 [52]	PD patients (N = 42, 22 M/20 F)	Examine reading difficulties in PD	Saccadic analysis during reading	Slower saccadic reading	Reading impairments linked to oculomotor dysfunction
9	Saccadic Dysfunction	Stuart, S., Lawson, R. A., Yarnall, A. J., Nell, J., Alcock, L., Duncan, G. W., ... & ICICLE-PD study group.	2019 [8]	PD patients (N = 75, 40 M/35 F)	Examine pro-saccades as predictor of cognitive decline	Saccadic eye-trackinng tasks	Prolonged saccadic latency predicts cognitive decline	Saccadic metrics correlate with executive dysfunction
10	Reading and Visual Impairments	Stock, L., Krüger-Zechlin, C., Deeb, Z., Timmermann, L., & Waldthaler, J.	2020 [53]	PD patients (N = 45, 22 M/23 F)	Investigate reading impairments in PD	Naturalistic reading tasks with eye-tracking	PD patients show reduced reading fluency	Reading difficulties linked to cognitive dysfunction
11	Pupillary Abnormalities	Kahya, M., Lyons, K. E., Pahwa, R., Akinwuntan, A. E., He, J., & Devos, H.	2021 [38]	PD patients (N = 50, 30 M/20 F)	Investigate pupillary responses to postural demands	Pupillometry and balance tasks	Abnormal pupillary reflex during postural adjustments	Pupil size linked to autonomic dysfunction
12	Fixation Instability	Tsitsi, P., Benfatto, M. N., Seimyr, G. Ö., Larsson, O., Svenningsson, P., & Markaki, I.	2021 [14]	PD patients (N = 55, 28 M/27 F)	Analyze fixation duration and pupil size as PD diagnostic tools	Pupillometry and eye-tracking	Shorter fixation duration, smaller pupils	Oculomotor markers for PD diagnosis
13	Pupillary abnormalities	Tsitsi, P., Benfatto, M. N., Seimyr, G. Ö., Larsson, O., Svenningsson, P., & Markaki, I.	2021 [14]	PD patients (N = 55, 28 M/27 F)	Investigate pupil size changes in PD	Eye-tracking and pupillometry	Reduce pupil dilatation in PD	Potential biomarker for cognitive decline
14	AI in PD diagnosis	Mei, J., Desrosiers, C., & Frasnelli, J.	2021 [43]	PD patients (N = 85, 50 M/35 F)	Review of machine learning for PD diagnosis	Literature review	Various AI models effective in PD classification	Potential for AI in automated diagnostics
15	Motor-Ocular Function	Fasano, A., Mazzoni, A., & Falotico, E.	2022 [37]	PD patients (N = 70, 40 M/30 F)	Asses reaching and grasping movements in PD	Oculomotor and motor coordination tests	Impaired visuomotor integration	Oculomotor deficits affect daily function
16	Saccadic Dysfunction	Kassavetis, P., Kaski, D., Anderson, T., & Hallett, M.	2022 [4]	PD patients (N = 50, 30 M/20 F)	Investigate eye movement disorders in PD	Clinical observation eye-tracking analysis	Hypometric saccades, increased latency	Saccadic impairments serve as early biomarkers
17	Smooth Pursuit Deficits	Fooken, J., Patel, P., Jones, C. B., McKeown, M. J., & Spering, M.	2022 [20]	PD patients (N = 60, 35 M/25 F)	Assess smoth pursuit impairments	Eye-tracking	Reduced pursuit gain, increased compensatory saccades	Deficits in motion tracking
18	Saccadic Dysfunction	Waldthaler, J., Vinding, M. C., Eriksson, A., Svenningsson, P., & Lundqvist, D.	2022 [21]	PD patients (N = 45, 25 M/20 F)	Examine neural correlates of impaired response inhibition	EEG and antisaccade tasks	Altered brain activity during saccade inhibition	Deficits in executive function
19	Saccadic Dysfunction	Fooken, J., Patel, P., Jones, C. B., McKeown, M. J., & Spering, M.	2022 [20]	PD patients (N = 60, 35 M/25 F)	Assess stimulus and task-specific preservation of ete movemets	Eye-tracking and neurocognitive assessments	Selective preservation of saccades in PD	Task-dependent variability in eye movements
20	AI in PD Diagnosis	Przybyszewski, A. W., Śledzianowski, A., Chudzik, A., Szlufik, S., & Koziorowski, D.	2023 [44]	PD patients (N = 90, 48 M/42 F)	Use machine learning to analyze eye movements in neurodegeneration	AI-based classification models	High accuracy in distinguishing PD from other disorders	Machine Learning improves PD diagnostic
21	Pupillary Abnormalities	Sun, Y. R., Beylergil, S. B., Gupta, P., Ghasia, F. F., & Shaikh, A. G.	2023 [11]	PD patients (N = 60, 33 M/27 F)	Analyze pupillary responses in PD	Pupillometry assessments	Reduced pupil dilatation linked to cognitive impairment	Potential biomarker for neurodegeneration
22	Smooth Pursuit Deficits	Swart, E. K., & Sikkema-de Jong, M. T.	2023 [33]	PD patients (N = 55, 28 M/27 F)	Examine effects of dopamine levels on smooth pursuit	Pharmacological eye-tracking	Dopamine modulates smooth pursuit accuracy	Dopaminergic treatment improves eye tracking
23	Saccadic Dysfunction	Riek, H. C., Brien, D. C., Coe, B. C., Huang, J., Perkins, J. E., Yep, R., & Munoz, D. P.	2023 [15]	PD patients (N = 55, 28 M/27 F)	Examine antisaccade behavior across neurodegenerative diases	Antisaccade eye-tracking tasks	Increased error rates in antisaccade tasks	Executive dysfunction correlates with saccadic impairments
24	VR- based Rehabilitation	Daniol, M., Hemmerling, D., Sikora, J., Jemiolo, P., Wodzinski, M., & Wojcik-Pedziwiatr, M.	2024 [12]	PD patients (N = 30, 18 M/12 F)	Assess VR applications in PD neurorehabilitation	Mixed reality and eye tracking	Improved visual search and spatial awareness	VR enhances motor-cognitive coordination
25	Ai in PD Diagnosis	Chudzik, A., Śledzianowski, A., & Przybyszewski, A. W.	2024 [10]	PD patients (N = 100, 55 M/45 F)	Assess AI and digital biomarkers in early PD detection	Machine learning analysis of eye-tracking data	High accuracy in early diagnosis	AI enhances diagnostic precision
26	Fixation Instability	Antoniades, C. A., & Spering, M.	2024 [28]	PD patients (N = 60, 32 M/28 F)	Investigate neurophysiological mechanisms of fixation instability	Eye tracking with neural recordings	Abnormal inhibitory control of fixational eye movements	Fixation instability as a biomarker for PD
27	Pupillary Abnormalities	Gibbs, M. C., Huxley, J., Readman, M. R., Polden, M., Bredemeyer, O., Crawford, T. J., & Antoniades, C. A.	2024 [6]	PD patients (N = 58, 31 M/27 F)	Analyze naturalistic eye movement tasks in PD	Pupillometry and real-word eye tracking	Reduced pupil dilation and impaired gaze control	Naturlistic tasks improve PD assessment
28	Ai in PD Diagnosis	Liao, X., Yao, J., Tang, H., Xing, Y., Zhao, X., Nie, D., ... & Li, G.	2024 [2]	PD patients (N = 100, 55 M/45 F)	Use AI-driven eye movement analysis for early PD detection	Machine learning on eye-tracking data	High predictive accuracy for early PD	AI-based eye-tracking enhances diagnostic precision
29	Motor-Ocular Function	Barbieri, F. A., Polastri, P. F., Barela, J. A., Bonnet, C. T., Brito, M. B., & Rodrigues, S. T.	2024 [31]	PD patients (N = 45, 26 M/19 F)	Investigate coupling of eye movements and postural stability	Eye tracking with balance assessments	PD patients compensate gaze instability with postural adjustments	Eye movement analisys informs fall risk assessment

## 4. Discussion

Eye-tracking technology has emerged as a powerful tool for understanding the neurophysiological mechanisms underlying Parkinson’s disease (PD), offering objective insights into motor, cognitive, and autonomic dysfunctions. Saccadic abnormalities, fixation deficits, and pupillary changes are characteristic features of PD disease progression. Measuring saccadic latency, accuracy, and inhibitory control deficits provides valuable insights into the integrity of the fronto-striatal network, making eye movement metrics relevant correlates of cognitive decline and executive dysfunction in patients with PD [54,55,56]. Given that executive impairments often emerge early in the disease course, eye-tracking assessments may serve as early warning signs of broader cognitive deterioration, including mild cognitive impairment (MCI) and Parkinson’s disease dementia (PDD). Machine learning approaches enhance the potential for automated diagnosis and monitoring by allowing AI-driven models to identify distinct oculomotor signatures and differentiate them from other neurodegenerative disorders, such as progressive supranuclear palsy (PSP) and Multiple System Atrophy (MSA). However, several key research priorities must be addressed to fully integrate eye-tracking technology into clinical practice. Standardizing eye-tracking tasks is crucial, as current methodologies vary significantly across studies, making it difficult to establish universally accepted diagnostic criteria. Developing consistent protocols for measuring saccadic dynamics, fixation control, and pupillary responses will ensure both reproducibility and clinical applicability.

Larger longitudinal studies are needed to validate oculomotor biomarkers, track changes over time, and determine their predictive value for motor and cognitive decline [57,58,59,60,61,62]. Expanding research efforts to include diverse patient populations will help refine oculomotor profiling techniques and establish eye tracking as a standard tool for PD monitoring.

Another critical area of exploration involves developing interventions that integrate gaze stabilization exercises, saccadic training, and VR-based feedback to improve visual exploration, attentional control, and motor coordination. Given the strong relationship between oculomotor control and executive function, designing therapeutic programs that complement pharmacological treatments can offer a multimodal approach to disease management.

These findings open promising avenues for integrating eye-tracking metrics into clinical assessments and designing tailored cognitive–motor rehabilitation protocols. Recent advances in the use of eye tracking and machine learning (ML) for the diagnosis and monitoring of Parkinson’s disease (PD) further support the relevance of the oculomotor markers discussed in this review. For example, Lukos et al. [63] and Zhou et al. [64] highlighted impaired saccadic control and eye–hand coupling in PD, extending the clinical interpretation of oculomotor dysfunction to real-world visuomotor tasks. These findings complement the markers identified in our synthesis, such as increased saccadic latency, fixation instability, and pupillary alterations, by demonstrating how they manifest in functionally meaningful contexts.

In parallel, studies such as those by Przybyszewski et al. [44], Brien et al. [65], and Bredemeyer et al. [43] have applied ML techniques to eye movement data, achieving promising classification accuracy for PD diagnosis. These approaches align with our findings on the consistency and diagnostic potential of specific oculomotor features, particularly in their capacity to serve as input variables for predictive models. Our review contributes to this growing body of evidence by identifying robust and recurrent markers that may support future ML-driven diagnostic tools and inform personalized rehabilitation strategies.

Future interventions should explore how training can enhance existing treatments, enabling a personalized and proactive approach to patient care. By harnessing the power of objective, data-driven assessments, clinicians and researchers can advance toward earlier diagnosis, targeted interventions, and continuous monitoring.

As technology progresses, wearable and mobile systems will enhance remote capabilities, enabling real-time tracking in naturalistic environments. The future of PD diagnosis and treatment lies in leveraging these innovations to develop precision medicine strategies, ensuring timely and individualized interventions that maximize functional independence and quality of life.

## 5. Conclusions

Eye tracking provides a reliable, cost-effective tool for early PD detection, cognitive assessment, and rehabilitation. Future research should focus on standardizing clinical protocols, validating predictive AI models, and integrating eye tracking into multimodal treatment strategies.

## Data Availability

No new data were created or analyzed in this study. Data sharing is not applicable to this article.
